# Studies on Curing Kinetics and Tensile Properties of Silica-Filled Phenolic Amine/Epoxy Resin Nanocomposite

**DOI:** 10.3390/polym11040680

**Published:** 2019-04-15

**Authors:** Ting Zheng, Xiaodong Wang, Chunrui Lu, Xiaohong Zhang, Yi Ji, Chengying Bai, Yiwen Chen, Yingjie Qiao

**Affiliations:** 1 School of Material Science and Chemical Engineering, Harbin Engineering University, Harbin 150001, China; zthappy1127@gmail.com (T.Z.); zhangxiaohong@hrbeu.edu.cn (X.Z.); memory330@126.com (Y.J.); chengyingbai@163.com (C.B.); ztqiuqiu@126.com (Y.C.); qiaoyingjie99@163.com (Y.Q.); 2 School of Materials Science and Engineering, Harbin Institute of Technology, Harbin 150001, China; luchunrui06@126.com

**Keywords:** cure kinetics, tensile strength, epoxy resin, nanocomposite

## Abstract

In this study, the curing kinetics of the phenolic amine/epoxy resin system were investigated by nonisothermal differential scanning calorimetry (DSC). The model-free isoconversional method of Ozawa–Flynn–Wall reveals a dependence of *E*_α_ (activation energy) on conversion (α), which interprets the autocatalytic curing reaction mechanism of the phenolic amine/epoxy resin system. Studies on the effects of nano-SiO_2_ particles on the tensile properties and tensile fracture face morphology of nanocomposites show that the uniform dispersion of SiO_2_ nanoparticles plays an important role in promoting the tensile performance of nanocomposites. Additionally, increases of 184.1% and 217.2% were achieved by adding 1.5% weight parts of nano-SiO_2_ in composites for the tensile strength and tensile modulus, respectively.

## 1. Introduction

As an important transportation equipment for offshore oil and gas exploitation, the safety of submarine oil–gas pipelines is related to the normal operation of offshore oil and gas exploitation system. Once the failure of submarine oil–gas pipelines occurs, it will not only cause the exploitation shutdown and the pollution of environment by crude oil, but also affect the normal production and life of an oil–gas supplier [1,2,3]. However, the traditional techniques widely used in repairing onshore pipelines are difficult to implement under water due to the complex undersea environment, long construction period, low repair speed and complex operational procedures [4,5,6]. Thus, it is urgent to develop a rapid and effective underwater repair technique to reduce loss and damage once oil–gas pipeline leaks.

Epoxy resins are very versatile materials with a wide range of applications based on their excellent chemical/corrosion resistance and mechanical properties [7]. For the past few decades, epoxy resins have been successfully applied in emergency repair and sealing of oil–gas pipelines due to the advantages of no welding, no production stopping, high construction safety, and low cost [8,9]. Epoxy resins’ composition, comprising phenolic amine curing agents prepared by the Mannich reaction, shows good, obtainable surface properties, in particular for coating, adhesively bonding and enhancing metallic and mineral substrates as an adhesive and sealant, and for producing moldings and sheet-like structures [10]. More importantly, this phenolic amine/epoxy resin system can be cured in a water-containing environment, thus it is considered to be a suitable material for underwater sealing and curing applications. It is well known that the physical properties of cured epoxy resins depend on the structure of their crosslinking network, curing extent, and the time and temperature of the curing reaction [11,12]. Such curing kinetics parameters related to network formation could provide substantial information concerning the final structure and properties of the epoxy resin network, and also the processability of epoxy resin. Therefore, the curing kinetics analysis of epoxy resin is essential to understand the structure–property–processing relationship for the preparation of high-performance composites [12]. However, there are few reports on the curing kinetics analysis of the phenolic amine/epoxy resin system until now.

In this work, dynamic differential scanning calorimetry (DSC) was used to study the curing kinetics of the phenolic amine/epoxy resin system. Detailed curing kinetics analysis was performed using the model-free isoconversional method of Ozawa–Flynn–Wall. Moreover, there are many unsaturated residual bonds and hydroxyl groups in different bonding states on the surface of nano-SiO_2_ particles, which is beneficial in improving interfacial bonding with the epoxy resin matrix in physical or chemical ways [13,14,15,16,17]. Therefore, nano-SiO_2_ particles were also used to further improve the mechanical properties of phenolic amine/epoxy resin composites. The influence of nano-SiO_2_ particle addition to epoxy resin on the tensile properties and fracture face morphology of the phenolic amine/epoxy resin system was also investigated. Based on the results, silica-filled phenolic amine/epoxy resin nanocomposites with good mechanical properties are a promising water-containing curing material for underwater speedy repair and sealing applications.

## 2. Materials and Methods

### 2.1. Materials

**Figure 1:**
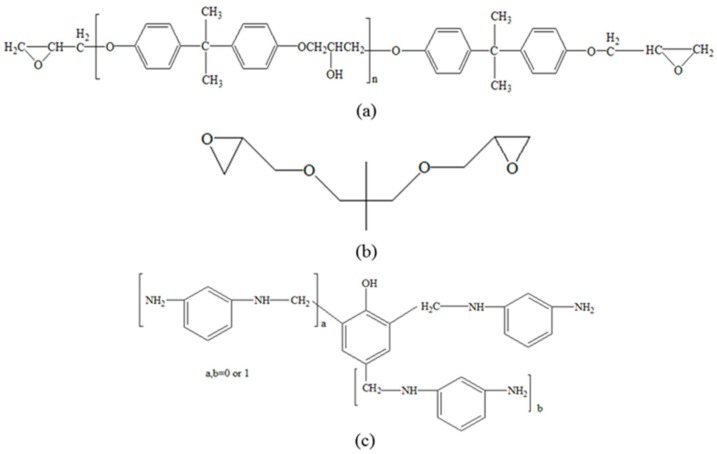
The structures of chemicals: (**a**) Diglycidyl ether of bisphenol A type epoxy resin (DGEBA), (**b**) neopentyl glycol diglycidyl ether (NGDE), and (**c**) phenolic amine 810.

A diglycidyl ether of bisphenol A type epoxy resin (DGEBA) provided by Nantong Xingchen Synthetic Material Co., Ltd. (Nantong, China) with an epoxy value of 0.51 was used in this study. Phenolic amine 810 provided by Changsha Chemical Industry Research Institute (Changsha, China) was applied to the hardener, and the neopentyl glycol diglycidyl ether (NGDE) supplied by Anhui Xinyuan Chemical Co., Ltd. (Huangshan, China) was added into the epoxy resin as a reactive diluent. Nano-SiO_2_ with a mean diameter of 12 nm was purchased from Evonik Degussa (Shanghai, China) and had an apparent density of 30 kg/m^3^ and a surface area of 200 ± 25 m^2^/g. The chemicals were used as received without further purification. The chemical structures of all materials used in this work are shown in Figure 1.

### 2.2. Dynamic DSC Analysis

Studies on the curing kinetics of the phenolic amine/epoxy resin system were carried out using a differential scanning calorimeter (DSC, 204 F1, NETZSCH, Selb, Germany). DGEBA, NGDE and phenolic amine 810 were mixed at room temperature based on their determined stoichiometric ratio. Samples of 5–10 mg were sealed in aluminum pans in respect to dynamic DSC scans, and heated up to 200 °C from a room temperature of 20 °C at a series of heating rates of 5, 10, 15, 20 and 25 °C/min, respectively.

### 2.3. Preparation of Nanocomposites

Nano-SiO_2_ particles were placed into a vacuum oven and heated at 100 °C for 2 h until the mass remained stable. The level of phenolic amine curing agent incorporation was based on the epoxy value of DGEBA according to the empirical formula of *q* = (1.3~1.4) × *k* × 43, where *q* and *k* are the amount of addition of phenolic amine curing agent per hundred of epoxy resin and the epoxy value, respectively [18]. According to this empirical formula, approximately 30 g of phenolic amine 810 was required for 100 g DGEBA. Epoxy-based reactive diluent NGDE can participate, together with DGEBA and phenolic amine 810, in polymerization and crosslinking reactions; therefore, 5 g more of NGDE was used in the phenolic amine/epoxy resin system.

In this paper, different formulations of DGEBA with phenolic amine 810 and NGDE were developed by varying the weight parts of SiO_2_ nanoparticles. The formulas employed in this study are listed in Table 1. The resultant materials were completely mixed by a mechanical stirrer and degassed with a vacuum pump to eliminate air bubbles. The bubble-free mixtures were then poured into preheated steel molds in an oven following curing at 40 °C for 3 h. Besides this, tensile test specimens were developed according to ASTM D638 tensile sample specification.

### 2.4. Tensile Test of Nanocomposites

A tensile test of all samples was carried out by a tensile tester (Instron 1196, Instron Corporation, Norwood, MA, USA) with a crosshead speed of 10 mm/min at room temperature. The tensile strength and tensile modulus of specimens were determined at the yield point and 0.5% strain, respectively. Five replicates were taken for each of the properties measured and the results given are the averages of five experimental values.

### 2.5. Scanning Electron Microscopy

The fracture surface morphology analysis of nanocomposites was performed using a scanning electron microscope (Phenom Pure, Phenom Scientific Instrument (Shanghai) Co. Ltd., Shanghai, China) at a 5 kV accelerating voltage. All specimen surfaces for SEM observation were coated with a thin gold film to protect fracture surfaces from beam damage and to prevent charge build-up.

## 3. Results

### 3.1. DSC Cure Characterization

**Figure 2:**
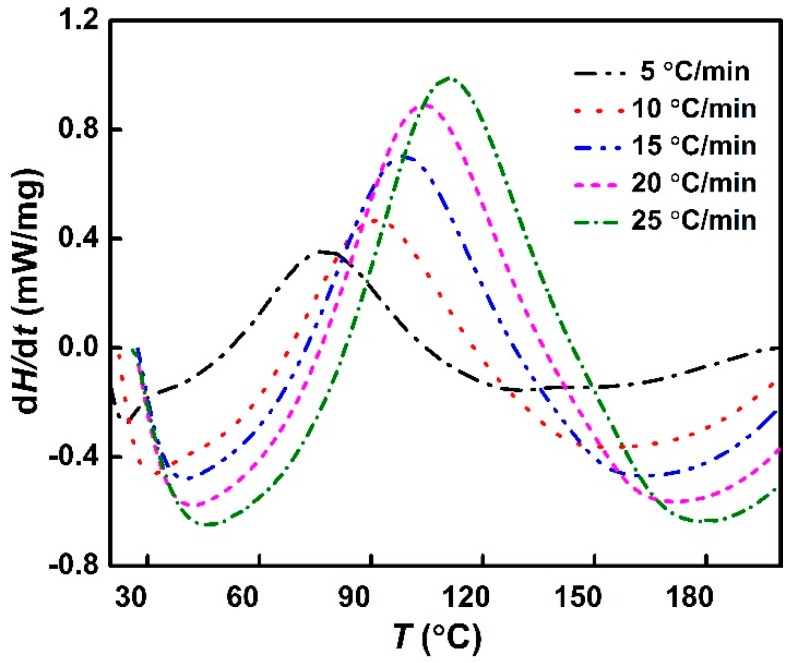
Differential scanning calorimetry (DSC) curves of the phenolic amine/epoxy resin system.

The typical DSC curves of DGEBA cured with 35 weight parts of 810 and 30 weight parts of NGDE displaying heat flow *dH/dt* against temperature *T* are shown in Figure 2. The information about the nature of the curing reaction, such as onset temperature *T*_i_, peak temperature *T*_p_, terminal temperature *T*_f_ and total exothermic reaction heat, can also be inferred from these curves, the results are listed in Table 2. It can be observed that, at any heating rate, there is a single broad peak. With an increase in heating rate, the exothermic peak temperature shifted to a higher value, because at a slow heating rate, the system took more time to get cured, and vice versa. In order to eliminate the influence of heating rate on curing system, *T*-*β* extrapolation method was used to calculate the gel point temperature (the initial temperature) *T*_gel_ and curing temperature (peak temperature) *T*_pc_ and post-curing temperature (termination temperature) *T*_fc_ at *β* = 0 [19]. *T*_i_, *T*_p_ and *T*_f_ were plotted against the heating rate *β*, and the curves were extrapolated to *β* = 0. Finally, the extrapolated results of *T*_i_, *T*_p_ and *T*_f_ of phenolic amine/epoxy resin system were 37.12, 72.39 and 106.35 °C, respectively. These results provide reference for the curing process temperature of phenolic amine/epoxy resin system.

### 3.2. Curing Kinetics Analysis

In nonisothermal DSC measurement, it is basically assumed that the measured heat flow, *dH/dt*, is proportional to the reaction rate, *dα*/*dt* [20]. For an unknown reaction mechanism, the reaction rate at a given time is considered to be the function of cure degree α [21], hence

(1) dα/dt=kf(α)

where *k* is the reaction rate constant and *f(α)* is a functional form of *α* depending on the reaction mechanism. *k* is assumed to be the Arrhenius form, so

(2) k=A exp(−E/RT)

where *A* is the pre-exponential factor, *R* is the universal gas constant, *E* is the value of activation energy and *T* is the absolute temperature. Equation (1) can be utilized to perform nonisothermal DSC experiments at different heating rates, β=dT/dt. By processing DSC curve data at different heating rates, it was easy to obtain the kinetic parameters above.

Peak temperature data for different heating rates from nonisothermal thermograms of each formulation were used to calculate the activation energy of the cure reaction, and the Kissinger and Ozawa methods were used for this calculation. According to the following formula,

(3) $$ln\left(\beta /T_{p}^{2}\right)=A\;exp\left(-E/RT\right)$$

The Kissinger method [22] is a linear plot of $ln\left(\beta /T_{p}^{2}\right)$ versus $1/T_{p}$. The values of activation energy *E* and pre-exponential factor *A* can be obtained by calculating the slope of linear fit and the intercept, respectively.

The Ozawa method [23] relates the logarithm of heating rate and the inverse of exothermic peak temperature, that is, ln(β) versus $1/T_{p}$. Therefore, the activation energy can be determined from the resultant slope:

(4) $$ln\left(\beta \right)=ln\left(AE/R\right)-lnF\left(\alpha \right)-5.331-1.052\left(E/RT_{p}\right)$$

(5) $$F\left(\alpha \right)=\overset{a}{\underset{0}{\int }}\frac{d\alpha }{f\left(\alpha \right)}$$

where *F*(*α*) is a constant function [24].

**Figure 3:**
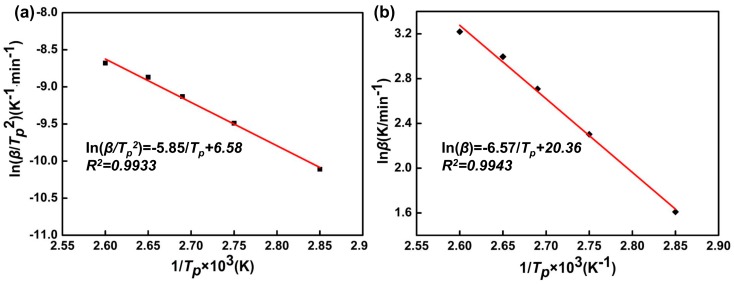
*E* determination of the phenolic amine/epoxy resin system by (**a**) the Kissinger method and (**b**) the Ozawa method.

Figure 3a,b gives linear fit plots of the methods of Kissinger and Ozawa, respectively. Activation energies obtained from Kissinger and Ozawa methods are 51.92 kJ/mol and 48.64 kJ/mol, respectively. The pre-exponential factor, *A*, can be determined by calculating the slope of the linear fit and the y-intercept through Figure 3a.

In addition, the simplest model used to describe the cure behavior of thermosets is the n-order curing reaction kinetic model generally (Equation (6)). The reaction order, *n*, of curing phenolic amine/epoxy resin system can be calculated from the Crane equation [25]:

*f* (α) = (1-α)^n^ (6)

(7) $$\frac{dln\beta }{d\left(\frac{1}{T_{p}}\right)}=-\left(\frac{E}{nR}+2T_{p}\right)$$

The reaction order, *n*, could be calculated by the slope of Figure 3b and the Crane equation. The average activation energy of Kissinger and Ozawa model methods and kinetic parameters are displayed in Table 3.

### 3.3. The Numerical Model of Curing Degree and Time

The actual curing reaction process can be predicted by using the kinetic parameters obtained above. Firstly, the equation of curing reaction rate cab be determined from Equations (1), (2) and (6):

(8) $$\frac{d\alpha }{\mathrm{dt}}=A\cdot \exp\left(-\frac{E}{\mathrm{RT}}\right){\left(1-\alpha \right)}^{n}$$

Introducing kinetic parameters in Equation (8), n-order reaction models of phenolic amine/epoxy resin composites can be defined as:

(9) $$\frac{d\alpha }{\mathrm{dt}}=4.22\times 10^{6}\cdot \exp\left(-\frac{6.05\times 10^{3}}{T}\right){\left(1-\alpha \right)}^{0.94}$$

Curing degree (or conversion) presents the degree of macro-reaction curing reaction and determines the curing performances of phenolic amine/epoxy resin composites. The dynamic model of curing degree of phenolic amine/epoxy resin composite was obtained by integrating the n-order kinetic model equation obtained above:

(10) $$\alpha =1-{\left[A\cdot \exp\left(-\frac{E}{RT}\right)\left(n-1\right)t+1\right]}^{\frac{1}{1-n}}$$

The curing reaction kinetics model of phenolic amine/epoxy resin composites can be established by substituting the parameters of *E, A* and *n* into integral Equation (11):

(11) $$\alpha =1-{\left[1-2.53\times 10^{5}\cdot \exp\left(-\frac{6.05\times 10^{3}}{T}\right)t\right]}^{16.67}$$

**Figure 4:**
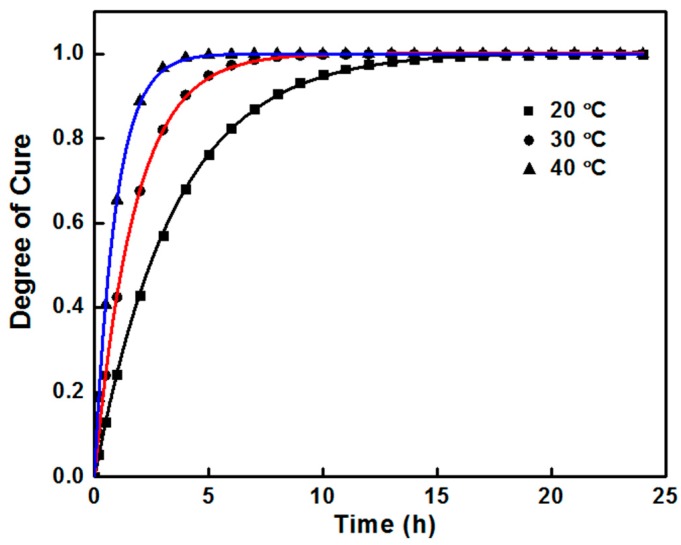
The degree of cure as a function of time at different cure temperature.

Formula (11) reflects the relationship of curing degree α, curing temperature *T* and curing time *t*. Through this formula, the relationship between α and *t* of phenolic amine/epoxy resin composites at different temperatures can be obtained. Therefore, the actual curing reaction process can be predicted. The curves of curing time and curing degree of phenolic amine/epoxy resin composites at different temperatures are presented in Figure 4. It shows that extending reaction time and increasing reaction temperature are two effective methods to achieve the same curing degree. In order to shorten the curing time, 40 °C and 3 h were selected as curing parameters in this paper. In this condition, phenolic amine/epoxy resin composites are cured almost completely.

### 3.4. Model-Free Isoconversional of Ozawa–Flynn–Wall

Epoxy resin curing is a complicated process due to the presence of gelation and vitrification, and therefore it is considered to be a multi-step reaction [26]. Isoconversional methods, which assume that the conversion is constant at the curing reaction DSC peak and independent of the several heating rates used, have been previously applied to the curing kinetics analysis of many other thermosetting systems and require no knowledge of the reaction model [27]. The above methods of Kissinger and Ozawa have the limitation of producing a single value of *E* for the whole process, which is a sign of a single-step process [28]. Model-free isoconversional methods can help to observe variations in apparent activation energy at different degrees of curing. The isoconversional method, more importantly, is able to explain the curing reaction mechanism by observing the change of apparent activation energy with conversion.

A more complete determination of *E* at any selected conversion can be received by adopting the popular isoconversional method of Ozawa–Flynn–Wall which relates conversion-dependent apparent activation energy, heating rate, and isoconversion temperature [29,30]. The basic equation of the Ozawa–Flynn–Wall method is

(12) $$ln\left(\beta \right)=ln\left(A\;E_{\alpha }/R\right)-lnG\left(\alpha \right)-5.331-1.052\left(E_{\alpha }/RT_{\alpha }\right)$$

(13) $$G\left(\alpha \right)=\overset{\alpha }{\underset{0}{\int }}\frac{d\alpha }{f\left(\alpha \right)}$$

where E_α_ is the value of apparent activation energy, *T*_α_ is the isoconversion temperature and *G*(*α*) is the integral conversion function.

**Figure 5:**
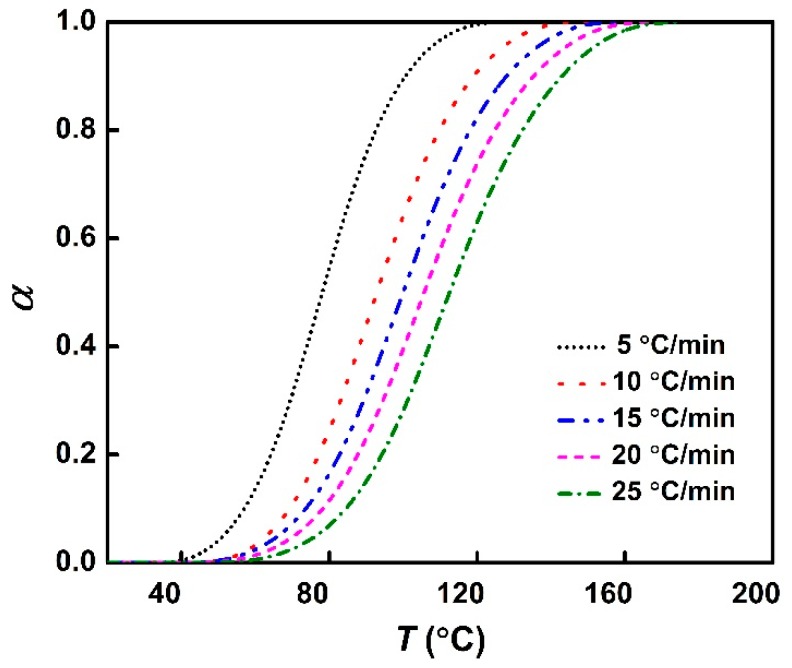
Conversion vs. temperature at different heating rates for the phenolic amine/epoxy resin system.

A transformation was performed to convert nonisothermal DSC scans into fractional conversion versus isoconversional temperatures at different heating rates. A representative of this transformation is shown in Figure 5 for the phenolic amine/epoxy resin system.

**Figure 6:**
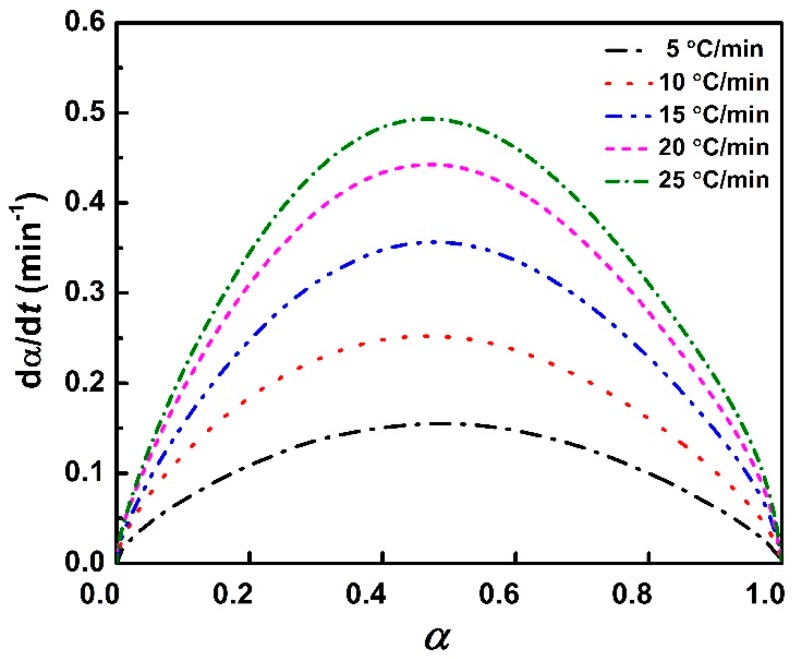
Reaction rate vs. conversion plot at different heating rates.

The curing reaction rate, *dα/dt = dH/dt/∆H*_total_, versus the conversion plot is also shown in Figure 6. The value of *dα/dt* increased to the maximum and then decreased to 0 with the increasing of α. Although increasing the heating rate promotes the reaction process, the maximum *dα/dt* values of reaction rate at a corresponding conversion rate is not basically changed. All α are in the range of 0.3–0.5, which is a character of an autocatalytic reaction [28].

**Figure 7:**
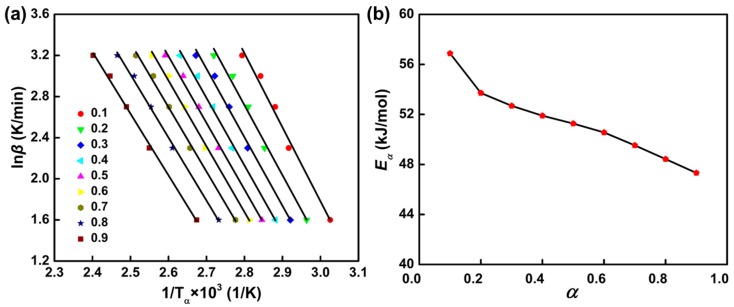
(**a**) Ozawa–Flynn–Wall method to determine apparent activation energy at different conversion rates. (**b**) Dependence of apparent activation energy on the conversion of the phenolic amine/epoxy resin system.

In order to understand apparent activation energy-conversion dependence, a plot of ln *β* as a function of 1/*T*_α_ obtained from DSC thermograms using various heating rates yields straight lines whose slope value is used to calculate the apparent activation energy (Figure 7a). Then, the activation energies calculated were plotted as shown in Figure 7b as a function of conversion. It is seen that E_α_ is highest at the beginning of curing, and then decreases with conversion throughout the entire curing process. The process of the curing reaction is autocatalytic because phenolic amine is an efficient hydrogen-bond donor molecule that promotes the ring opening of the epoxy group in the same way as the hydroxyl group of epoxy resin, leading to an auto-accelerating effect on the curing reaction and a decrease in apparent activation energy [31,32]. In the initial stages of reaction, the generated hydroxyl groups are less and the catalytic effect is not obvious, thus the reaction activation energy is higher. As the reaction continues, the generated hydroxyl groups increase to promote the catalytic reaction effectively, the activation energy will be decreased. In addition, the temperature of the curing system increases and viscosity is reduced during the process of the curing reaction. These also accelerate the diffusion rate and reduce the activation energy [33].

**Figure 8:**
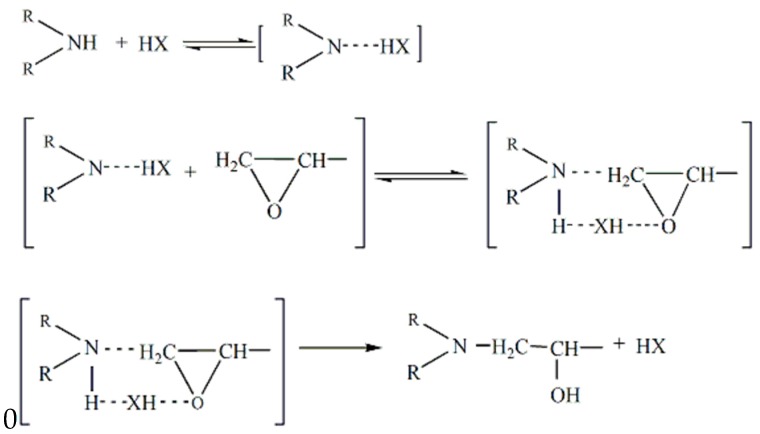
Schematic illustration of a trimolecular transition state amine–epoxy–hydroxyl.

However, it is noteworthy that the *E_α_* varies in a narrower range (about 10 kJ/mol) with respect to *α* compared to the range reported for other epoxy-amine systems [34]. Values around 50 kJ/mol were obtained, which are in good agreement with Kissinger and Ozawa model-fitting kinetics values and literature data for the autocatalytic reaction of an epoxy–amine system [35]. The rate-determining steps for autocatalytic reactions correspond to the formation of a trimolecular transition state amine–epoxy–hydroxyl, maybe as shown in Figure 8, where HX represents a hydrogen-bond donor.

### 3.5. SiO_2_/Epoxy Nanocomposites

**Figure 9:**
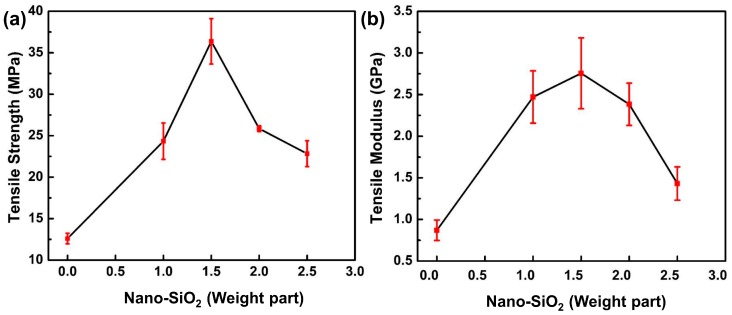
(**a**) Tensile strength and (**b**) tensile modulus vs. nano-SiO_2_ content for nanocomposites.

The tensile strength obtained for SiO_2_/epoxy nanocomposites with different weight parts of nano-SiO_2_ particles in nanomaterials system is plotted in Figure 9. It shows that the tensile strength of nanocomposites increases with the addition of SiO_2_ nanoparticles up to 1.5 weight parts, where the value of tensile strength reaches its maximum and then decreases with further additions. The tensile strength of nanocomposites with 1.5 weight parts of nano-SiO_2_ particles increased remarkably up to 36.38 MPa in comparison with the pure epoxy resin with a tensile strength of 12.60 MPa.

A similar tendency for the tested parameters in terms of the variation of nano-SiO_2_ particles content was observed for the tensile modulus of nanocomposites, as shown in Figure 9b. The tensile modulus was enhanced significantly with 1.5 weight parts of SiO_2_ nanoparticles in nanomaterials system, and the value of the tensile modulus increased by 217.2%.

The dispersion ability of SiO_2_ nanoparticles in epoxy resin is believed to be responsible for the increase-then-decrease phenomenon in terms of its tensile properties [36]. Nano-SiO_2_ particles are shown to have a strong agglomerating trend, so that the homogeneous dispersion of SiO_2_ nanoparticles has been considered to be a difficult and important process [37]. A good dispersion, however, can be achieved by surface modification and appropriate processing conditions [38,39]. In this work, nano-SiO_2_ particles were sufficiently premixed with NGDE and placed for more than 8 h at room temperature. Of course, a reasonably good distribution of SiO_2_ nanoparticles can be achieved at a low content.

**Figure 10:**
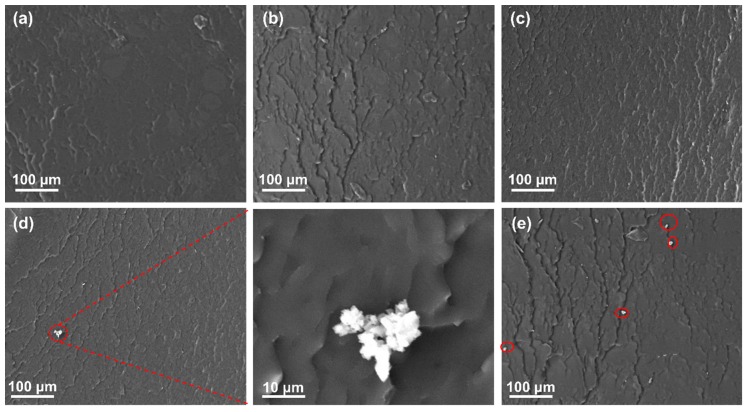
SEM micrographs of fractured nanocomposites filled with different weight parts nano-SiO_2_ (**a**) 0, (**b**) 1.0, (**c**) 1.5, (**d**) 2.0 and (**e**) 2.5.

Figure 10 exhibits typical SEM micrographs of tensile fracture surfaces of nanocomposites containing different weight parts of nano-SiO_2_ particles. Noticeably, an amount of wrinkles (Figure 10b,c) appear on the fracture surfaces of nanocomposites with 1.0 and 1.5 weight parts of nano-SiO_2_ particles in contrast with the relatively smooth one (Figure 10a), indicating that massive new surfaces were created under tensile loading. This should maximize the interaction between SiO_2_ nanoparticles and epoxy resin, and much more energy can be dissipated through these new surfaces, leading to significant improvement of the tensile properties of nanocomposites with 1.0 and 1.5 weight parts nano-SiO_2_ particles (Figure 9).

Further additions of SiO_2_ nanoparticles generally make uniform dispersion difficult, resulting in a weak-bonding interface between the filler and matrix. We can observe that small aggregates begin to appear on the fracture surfaces of nanocomposites with 2.0 weight parts nano-SiO_2_ particles as Figure 10d and its enlarged SEM picture displayed. The nanocomposite with 2.5 weight parts nano-SiO_2_ particles shows even more clusters (as indicated by the red circles in Figure 10e). This may be the main and a reasonable interpretation of the reduction on the tensile properties of nanocomposites with 2.0 and/or 2.5 weight parts nano-SiO_2_ particles.

## 4. Conclusions

The activation energies obtained by Kissinger and Ozawa methods are 51.92 and 48.64 kJ/mol, respectively. The experimental dependence of *E*_α_ on α determined from the model-free isoconversional kinetics analysis method of Ozawa–Flynn–Wall can be adequately interpreted in terms of the autocatalytic curing reaction mechanisms of the phenolic amine/epoxy resin system.

The effects of the nano-SiO_2_ particles content on the tensile properties and fracture surface morphology of nanocomposites were also investigated. Studies of the morphology of fracture faces suggest that a uniform distribution of SiO_2_ nanoparticles is essential in promoting tensile properties. The tensile performance of nanocomposites reached its optimum at 1.5 weight parts of SiO_2_ nanoparticle addition, where 184.1% and 217.2% increases were achieved for tensile strength and tensile modulus compared to the pure epoxy resin, respectively.

## Figures and Tables

**Table 1:** Weight parts of each materials in phenolic amine/epoxy resin system for tensile performance testing.

Samples	Component and Content			
	DGEBA	810	NGDE	Nano-SiO_2_
1	100	35	30	0
2	100	35	30	1.0
3	100	35	30	1.5
4	100	35	30	2.0
5	100	35	30	2.5

**Table 2:** Data from the analysis of DSC measurements at different heating rates.

Heating Rate (°C/min)	*T*_i_/°C	*T*_p_/°C	*T*_f_/°C	∆*H*_total_ (J/g)
5	40.73	77.41	112.6	247.1
10	52.74	91.13	134.7	222.5
15	58.16	98.50	146.0	184.6
20	62.30	103.7	153.2	177.9
25	68.08	111.1	162.4	162.5

**Table 3:** Kinetic parameters determined by fitting nonisothermal DSC measurements.

*E* (kJ·mol^−1^)	n	A (min^−1^)
50.28	0.94	4.22 × 10^6^
